# Plumage condition in laying hens: genetic parameters for direct and indirect effects in two purebred layer lines

**DOI:** 10.1186/1297-9686-46-33

**Published:** 2014-05-29

**Authors:** Tessa Brinker, Piter Bijma, Jeroen Visscher, T Bas Rodenburg, Esther D Ellen

**Affiliations:** 1 Animal Breeding and Genomics Centre, Wageningen University, P.O. Box 338, 6700 AH, Wageningen, The Netherlands; 2 Institut de Sélection Animale B.V., Hendrix Genetics Company, P.O. Box 114, 5830 AC, Boxmeer, The Netherlands; 3 Behavioural Ecology Group, Wageningen University, P.O. Box 338, 6700 AH, Wageningen, The Netherlands

## Abstract

**Background:**

Feather pecking is a major welfare issue in laying hen industry that leads to mortality. Due to a ban on conventional cages in the EU and on beak trimming in some countries of the EU, feather pecking will become an even bigger problem. Its severity depends both on the victim receiving pecking and on its group mates inflicting pecking (indirect effects), which together determine plumage condition of the victim. Plumage condition may depend, therefore, on both the direct genetic effect of an individual itself and on the indirect genetic effects of its group mates. Here, we present estimated genetic parameters for direct and indirect effects on plumage condition of different body regions in two purebred layer lines, and estimates of genetic correlations between body regions.

**Methods:**

Feather condition scores (FCS) were recorded at 40 weeks of age for neck, back, rump and belly and these four scores were added-up into a total FCS. A classical animal model and a direct–indirect effects model were used to estimate genetic parameters for FCS. In addition, a bivariate model with mortality (0/1) was used to account for mortality before recording FCS. Due to mortality during the first 23 weeks of laying, 5363 (for W1) and 5089 (for WB) FCS records were available.

**Results:**

Total heritable variance for FCS ranged from 1.5% to 9.8% and from 9.8% to 53.6% when estimated respectively with the classical animal and the direct–indirect effects model. The direct–indirect effects model had a significantly higher likelihood. In both lines, 70% to 94% of the estimated total heritable variation in FCS was due to indirect effects. Using bivariate analysis of FCS and mortality did not affect estimates of genetic parameters. Genetic correlations were high between adjacent regions for FCS on neck, back, and rump but moderate to low for belly with other regions.

**Conclusion:**

Our results show that 70% to 94% of the heritable variation in FCS relates to indirect effects, indicating that methods of genetic selection that include indirect genetic effects offer perspectives to improve plumage condition in laying hens. This, in turn could reduce a major welfare problem.

## Background

Feather pecking (FP) is a major welfare issue in commercial laying hens. Depending on the severity of the pecking it can result in feather loss or damage, or skin or muscle injuries [1]. If the latter results in death, FP is referred to as cannibalism [2]. When hens are not beak trimmed, the incidence of cannibalistic FP is higher in non-cage systems than in cage systems [3]. Since the beginning of 2012, cage systems have been prohibited in the European Union and the problem of FP is expected to increase [4]. Therefore, it is necessary to find a solution to prevent or reduce FP, especially when beak treatments are banned or will be banned in the future.

FP is a multi-factorial problem caused by both animal-related and environmental factors [5]. A common procedure to limit the consequences of FP is beak trimming. There are plans to ban beak trimming since this procedure has welfare implications, such as chronic pain [6]. In some European countries, beak trimming is already prohibited or regulated [7]. Other management solutions could be feed-related [8]. Furthermore, it has been shown that FP behaviour can be influenced by light intensity [9], provision of floor litter [10], group size [11], and stocking density [12]. So far, there are no management solutions that can completely prevent FP. An additional measure to reduce FP is genetic selection [13,14].

FP is a social interaction phenomenon, that involves both the victim that receives the pecking and its group mates that inflict the pecking [15-18]. When traits are affected by social interactions among group members, the genetic effects that underlie individual phenotypes can be partitioned into a direct genetic effect (DGE) of the genotype of the individual itself, and the indirect genetic effects (IGE) of the genotypes of its group mates [19-24]. IGE can contribute to the heritable variation in a trait. For example, in laying hens showing cannibalistic behaviour, IGE contribute 33% to 87% of the total heritable variation in survival time [25,26]. Genetic selection for survival time, using a selection method that takes IGE into account, could reduce FP behaviour. Unfortunately, survival time is only known at the end of the laying period. Therefore, it is necessary to define a trait that can be collected early in the laying period and that is correlated with FP behaviour.

To measure FP, behavioural observations are needed. However, several authors have used a feather condition score (FCS) to assess plumage condition as an alternative to behavioural observations [5,27,28]. Damage to the plumage is strongly related to the incidence of severe FP behaviour [28]. One disadvantage of using FCS instead of direct observations of pecking behaviour is that one can only detect the victim of FP and not the animal that actually inflicts the pecking. However, using methods that take IGE into account allows us to estimate both the breeding value for pecker-effect (the IGE) and for the victim-effect (the DGE) in group-housed laying hens [24,29]. Estimating a breeding value for the pecker-effect is less accurate than using behavioural observations but, in animal breeding, it is not feasible to record a behavioural observation for each individual but it is possible to estimate DGE and IGE for each individual.

So far, most studies that focus on the plumage condition of laying hens have ignored the effect of group mates. However, it is expected that IGE contribute significantly to the heritable variation of plumage condition in laying hens, especially in the case of damaging behaviour such as FP. To improve plumage condition in laying hens, knowledge of the genetic parameters for both direct and indirect effects is required.

In the present study, genetic parameters of plumage condition in laying hens were estimated using a classical animal model and a model that combined both direct and indirect genetic effects. Plumage condition was measured on four body regions; neck, back, rump and belly using the FCS. In addition, genetic correlations between the different body regions were estimated.

## Methods

For this study, data from the experiment that is described in Ellen et al. [25] were used except that FCS were used instead of survival time data. The main characteristics are summarized below. Further details are in Ellen et al. [25].

### Population and pedigree

Data were collected under control of Institut de Sélection Animale B.V., the layer breeding division of Hendrix Genetics. Hendrix Genetics complies with the Dutch law on animal wellbeing. Data on two purebred White Leghorn layer lines were provided by the Institut de Sélection Animale B.V.. The two lines were coded W1 and WB. Data from line WF were not used in this study because fewer observations were available for this line and its mortality due to cannibalism was low (in comparison with the other two lines), which would lead to inaccurate genetic parameters for direct and indirect effects of survival time [25].

Within a line, sires and dams were mated at random. Matings were done in two batches with a six-month time period between the two batches. Sires used for both batches were largely the same (89% for line W1 and 94% for line WB), while dams were all different. For each batch, sires (36 of line W1 and 35 of line WB) were mated to approximately eight dams each, and each dam contributed on average 12.3 female offspring. For both lines, observations from a single generation were used. Chickens of both lines were hatched in two batches and each batch consisted of four consecutive age groups that differed by a two-week period each. All 12 192 chickens had intact beaks.

### Housing

When the hens were on average 17 weeks old, they were transported to two laying houses with traditional four-bird-battery cages. The two batches were each placed in separate laying houses, termed 1 and 2. In both laying houses, the 17-week-old hens were randomly allocated to laying cages, with four birds of the same line and age in a cage. The individuals making up a cage were combined at random, without taking size of the hens into account. In both laying houses, cages were grouped into eight double rows. Each row consisted of three levels (top, close to the light; middle; and bottom). A feeding trough was in front of the cages and each pair of back-to-back cages shared two drinking nipples. A standard commercial layer diet and water were provided *ad libitum*.

In both laying houses, the hens started with a light period of 9 hours/day. Every week the light period was increased by 1 hour until 16 hours/day, when the hens were on average 26 weeks of age.

### Data

Plumage condition was measured at 40 weeks of age on all hens alive. It was measured at eight time points, each separated by a two-week period, starting with the oldest hens, so that all birds were measured at the same age. To quantify plumage condition, the feather condition score (FCS) described in Bilčik and Keeling [28] was used, as modified by Uitdehaag et al. [30]. The body of the hen was divided into four regions: neck, back, rump, and belly. These body regions were chosen because they are expected to be the regions to receive the largest number of pecks and plumage condition in those regions is less affected by abrasion [28]. Each body region was inspected and given a score from 0 (intact feathers) to 5 (completely denuded area). For further analysis, observations with a score 0 or 1 were combined into the score 1 class. FCS were recorded by four persons. Before performing the FCS, a single set of 153 birds were scored by the four persons to estimate the correlation between scores of different persons (between-observer correlation). The between-observer correlation ranged from 0.84 to 0.94 for neck, back, and rump but from 0.66 to 0.83 for belly (ED Ellen, unpublished data).

All hens were observed daily. Dead hens were removed, and wing band number, cage number, and cause of death were recorded. Cause of death was determined subjectively without dissection. Thus for all hens in the dataset, their status alive (0) or dead (1) was known at the time of feather scoring. A total of 12 192 hens were present in the dataset composed of 5920 hens of line W1 and 6272 hens of line WB. Due to mortality during the first 23 weeks of laying, FCS were unavailable for 9.4% of the W1 hens and for 18.9% of the WB hens, which resulted in 5363 FCS records for line W1 and 5089 for line WB.

### Data analysis

#### Model

A preliminary data analysis was performed using the SAS statistical program [31]. The GLM procedure was used to identify significant fixed effects to be included in the model for subsequent analysis. Analysis of FCS was done for each line and body region separately. The four body regions were summed into a total FCS, which was also analysed. The most significant fixed effects identified were the interaction between laying house-row-level and the person carrying out the scoring. Age and batch were fully confounded with laying house and row, and therefore not included in the model.

A linear animal model was used in ASReml to estimate genetic parameters for FCS [32]. First, genetic parameters were estimated by using a classical animal model,

(1) y=Xb+Za+Vc+e,

where **y** is a vector of observed FCS; **b** is a vector of fixed effects, with incidence matrix **X** linking observations to the fixed effects; **a** is a vector of the breeding values, with incidence matrix **Z** linking the observations on individuals to their breeding value; **c** is a vector of independent random cage effects, with incidence matrix **V** linking the observations to the random cage effect; and **e** is a vector of random residuals. The variance structure of the model terms are: $\mathrm{var}\left[a\right]=A\sigma_{A}^{2}$, $\mathrm{var}\left[c\right]=I\sigma_{c}^{2}$, and $\;\mathrm{var}\left[e\right]=I\sigma_{e}^{2}$. Matrix **A** is the matrix of additive genetic relationships between individuals based on five generations of pedigree, $\sigma_{A}^{2}$ the genetic variance, **I** an identity matrix, $\sigma_{c}^{2}$ the cage variance, and $\sigma_{e}^{2}$ the residual variance. To avoid pedigree errors, hens with an unknown or double identification were coded as having an unknown pedigree (*n* = 63). The observations on these hens were included in the analysis to better estimate fixed effects.

Second, genetic parameters were estimated for both the direct and indirect genetic effects using a direct–indirect effects model [23,24],

(2) $$y=\mathrm{Xb}+Z_{D}a_{D}+Z_{S}a_{S}+\mathrm{Vc}+e,$$

where **a**_D_ is a vector of direct breeding values, with incidence matrix **Z**_D_ linking observations on individuals to their direct breeding value, **a**_S_ is a vector of indirect breeding values, with incidence matrix **Z**_S_ linking observations on individuals to the indirect breeding values of their group mates (the other three individuals in the same cage; see [24]). The covariance structure of the genetic terms is $\mathrm{var}\left[\begin{array}{c} a_{D}\\ a_{S} \end{array}\right]=C⊗A$, where ⊗ is the Kronecker product of matrices, and $C=\left[\begin{array}{cc} \sigma_{A_{D}}^{2} & \sigma_{A_{\mathrm{DS}}}\\ \sigma_{A_{\mathrm{DS}}} & \sigma_{A_{S}}^{2} \end{array}\right]$, where $\;\sigma_{A_{D}}^{2}$ is the direct genetic variance, $\;\sigma_{A_{S}}^{2}$ is the indirect genetic variance, and $\sigma_{A_{\mathrm{DS}}}$ is the direct–indirect genetic covariance.

#### Model comparison

The classical animal model (Equation 1) and the direct–indirect effects model (Equation 2) were statistically compared using a log-likelihood ratio test. The classical animal model was compared with a model without random effects (null model) and a model with only a random cage effect to test additive genetic variance. The direct–indirect effects model was compared with the classical animal model to test the indirect genetic (co)variance.

#### Heritable variation

In the classical animal model, the heritability is the ratio of heritable variance ($\sigma_{A}^{2}$) and phenotypic variance ($\sigma_{P}^{2}$):

(3) $$h^{2}=\frac{\sigma_{A}^{2}}{\sigma_{P}^{2}}.$$

In the direct–indirect effects model, the total heritable variance ($\sigma_{\mathrm{TBV}}^{2}$) available for response to selection is $\sigma_{\mathrm{TBV}}^{2}=\sigma_{A_{D}}^{2}+2\left(n-1\right)\sigma_{A_{\mathrm{DS}}}+{\left(n-1\right)}^{2}\sigma_{A_{S}}^{2}$, where *n* is the number of individuals in a group [22]. Phenotypic variance is $\sigma_{P}^{2}=\sigma_{A_{D}}^{2}+\left(n-1\right)\sigma_{A_{S}}^{2}+\sigma_{e}^{2}$. The term (*n* - 1) in both expressions refers to the  *n* - 1 group mates with which the individual interacts. For socially affected traits, the ratio of total heritable variance and phenotypic variance (*T*^2^) is:

(4) $$T^{2}=\frac{\sigma_{\mathrm{TBV}}^{2}}{\sigma_{P}^{2}}.$$

A comparison of *h*^2^ and *T*^2^ reflects the impact of IGE on heritable variation.

#### Selection bias

Estimates of genetic parameters for single traits such as FCS can be biased when the data represent a selected subset of the population [33]. In lines W1 and WB, the percentage of animals that died before FCS was recorded was 9.4% and 18.9%, respectively. These dead animals can bias the estimated genetic parameters, since they are expected to have a higher FCS (more damage). Such selection bias can be reduced by using multiple-trait analysis [33]. For this reason, a bivariate analysis (Equation 5), including both FCS and mortality at 40 weeks of age (0/1) was applied to both the classical animal model and the direct–indirect effects model. In the bivariate analysis, the model for mortality at 40 weeks of age (0/1) included only a DGE, since models that included both DGE and IGE failed to converge.

#### Genetic correlations

Genetic correlations between the different body regions were estimated using a classical animal and the direct–indirect effects models. To estimate genetic correlations using the direct–indirect effects model, the bivariate model of Peeters et al. [26] was used,

(5) $$\begin{array}{l} \left[\begin{array}{c} y_{1}\\ y_{2} \end{array}\right]=\left[\begin{array}{cc} X_{1} & 0\\ 0 & X_{2} \end{array}\right]\left[\begin{array}{c} b_{1}\\ b_{2} \end{array}\right]+\left[\begin{array}{cc} Z_{1_{D}} & 0\\ 0 & Z_{2_{D}} \end{array}\right]\left[\begin{array}{c} a_{1_{D}}\\ a_{2_{D}} \end{array}\right]\\ \;+\left[\begin{array}{cc} Z_{1_{S}} & 0\\ 0 & Z_{2_{S}} \end{array}\right]\left[\begin{array}{c} a_{1_{S}}\\ a_{2_{S}} \end{array}\right]+\left[\begin{array}{cc} V_{1} & 0\\ 0 & V_{2} \end{array}\right]\\ \;\times \left[\begin{array}{c} c_{1}\\ c_{2} \end{array}\right]+\left[\begin{array}{c} e_{1}\\ e_{2} \end{array}\right] \end{array}$$

where subscripts 1 and 2 denote FCS on two different body regions, e.g. neck and back. All other terms are the same as for Equation 2. The corresponding covariance structure of the genetic terms is

$$\mathrm{var}\left[\begin{array}{c} a_{1_{D}}\\ a_{2_{D}}\\ a_{1_{S}}\\ a_{2_{S}} \end{array}\right]=C⊗A,$$

with

$$C=\left[\begin{array}{cccc} \sigma_{A_{1_{D}}}^{2} & \sigma_{A_{12_{D}}} & \sigma_{A_{1_{\mathrm{DS}}}} & \sigma_{A_{1_{D}2_{S}}}\\ \sigma_{A_{12_{D}}} & \sigma_{A_{2_{D}}}^{2} & \sigma_{A_{2_{D}1_{S}}} & \sigma_{A_{2_{\mathrm{DS}}}}\\ \sigma_{A_{1_{\mathrm{DS}}}} & \sigma_{A_{2_{D}1_{S}}} & \sigma_{A_{1_{S}}}^{2} & \sigma_{A_{12_{S}}}\\ \sigma_{A_{1_{D}2_{S}}} & \sigma_{A_{2_{\mathrm{DS}}}} & \sigma_{A_{12_{S}}} & \sigma_{A_{2_{S}}}^{2} \end{array}\right].$$

Thus there are four genetic variances and six genetic covariances; $\sigma_{12_{D}}$ and $\sigma_{12_{S}}$ are the direct and indirect genetic covariances between two body regions, $\sigma_{1_{\mathrm{DS}}}$ and $\sigma_{2_{\mathrm{DS}}}$ are the genetic covariances between direct and indirect effects for one of the body regions, and $\sigma_{1_{D}2_{S}}$ and $\sigma_{2_{D}1_{S}}$ are the genetic covariances between the direct effect of one body region and the indirect effect of another body region. For all body regions and lines, genetic correlations were estimated between the DGE ($r_{12_{D}}$), the IGE ($r_{12_{S}}$), and the TBV (total breeding value) ($r_{12_{T}}$). Correlation $r_{12_{T}}$ depends on the total heritable variance within body regions ($\sigma_{\mathrm{TB}V_{1}}^{2}$ and $\sigma_{\mathrm{TB}V_{2}}^{2}$) and the total genetic covariance between body regions ($\sigma_{\mathrm{TB}V_{12}}$), and is given by $r_{12_{T}}=\frac{\sigma_{A_{12_{D}}}+\left(n-1\right)\sigma_{A_{1_{D}2_{S}}}+\left(n-1\right)\sigma_{A_{2_{D}1_{S}}}+{\left(n-1\right)}^{2}\sigma_{A_{12_{S}}}}{\sqrt{\left(\sigma_{A_{1_{D}}}^{2}+2\left(n-1\right)\sigma_{A_{1_{\mathrm{DS}}}}+{\left(n-1\right)}^{2}\sigma_{A_{1_{S}}}^{2}\right)\left(\sigma_{A_{2_{D}}}^{2}+2\left(n-1\right)\sigma_{A_{2_{\mathrm{DS}}}}+{\left(n-1\right)}^{2}\sigma_{A_{2_{S}}}^{2}\right)}}$[26].

In the bivariate analyses, the random cage effect and residual were also allowed to be correlated between body regions.

## Results and discussion

### Feather score

**Figure 1:**
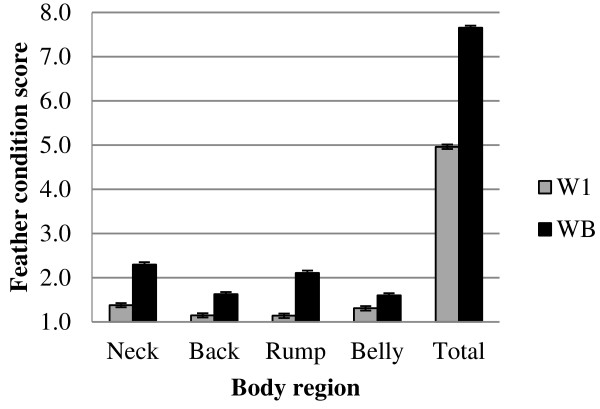
FCS of each body region and total FCS for two lines (W1, WB), with corresponding SE.

Average plumage condition differed between body regions (Figure 1) and between the two lines (P < 0.001). Overall, line WB yielded the highest average FCS (worst plumage condition), ranging from 1.6 (belly) to 2.3 (neck). Average FCS by region for line W1 ranged from 1.1 (rump) to 1.4 (neck). This is in line with results of Ellen et al. [25], who found that average survival was lowest in line WB. Overall, the plumage condition was worst for the neck region and best for the back region. This is in contradiction with results of Bilčik and Keeling [28] who used the same scoring method, but, in their study, hens were kept in groups of 15 birds. They found that plumage condition for the hybrid layer line Hisex white was worst for the belly region, and best for the neck and back regions. In addition, they showed that, although belly had the worst plumage condition, most pecks were targeted at the rump and back. These discrepancies with our results might be due to differences in line, age, and housing conditions such as density, size of groups, and light intensity. One major difference between the studies is that Bilčik and Keeling birds were housed in floor pens, while in the present study birds were housed in conventional cages. In conventional cages, much higher levels of abrasion of neck feathers are observed, due to contact with the cage door while feeding. The abrasion of neck feathers could stimulate the feather pecking behaviour of group mates, which can result in higher levels of neck damage.

The FCS was significantly different between the two laying houses, except for belly (P = 0.055). In laying house 2, back and rump had the lowest FCS, whereas in laying house 1 neck had the lowest FCS. Line WB had the worst plumage condition in both laying houses. Furthermore, significant differences in FCS between the three levels of each row and between the corridors were found (Table 1), except for the effect of level on rump, belly, and total FCS (P > 0.05). Overall, the lowest FCS (best plumage condition) were recorded on birds located on the bottom level, whereas the highest FCS were recorded on birds on the top and middle levels. Upper levels had higher light intensities, which stimulate FP behaviour and thus result in higher FCS (worst plumage condition) [5,25]. Overall, the highest FCS were observed for rows 7 and 8 without any clear explanation.

**Table 1:** Number of hens (n) and least square means (±SE) for FCS

	**n**	**Neck**	**Back**	**Rump**	**Belly**	**Total**
Laying house						
1	7376	1.80 ± 0.01	1.41 ± 0.01	1.75 ± 0.01	1.44 ± 0.01	6.40 ± 0.03
2	4816	1.89 ± 0.02	1.36 ± 0.01	1.50 ± 0.02	1.47 ± 0.01	6.21 ± 0.05
Level						
Top^1^	2400	1.87 ± 0.02	1.39 ± 0.02	1.64 ± 0.02	1.46 ± 0.02	6.36 ± 0.06
Middle	4772	1.85 ± 0.01	1.41 ± 0.01	1.64 ± 0.01	1.44 ± 0.01	6.33 ± 0.04
Bottom	5020	1.81 ± 0.01	1.36 ± 0.01	1.60 ± 0.01	1.46 ± 0.01	6.23 ± 0.04
Row						
1	1096	1.69 ± 0.03	1.19 ± 0.03	1.29 ± 0.03	1.32 ± 0.02	5.49 ± 0.09
2	1316	1.57 ± 0.03	1.15 ± 0.02	1.28 ± 0.03	1.41 ± 0.02	5.41 ± 0.08
3	1524	1.56 ± 0.02	1.25 ± 0.02	1.41 ± 0.03	1.35 ± 0.03	5.58 ± 0.07
4	1648	1.60 ± 0.02	1.23 ± 0.02	1.38 ± 0.02	1.27 ± 0.03	5.48 ± 0.07
5	1632	1.83 ± 0.02	1.42 ± 0.02	1.79 ± 0.02	1.52 ± 0.03	6.57 ± 0.07
6	1616	1.93 ± 0.02	1.45 ± 0.02	1.79 ± 0.03	1.65 ± 0.03	6.82 ± 0.07
7	1700	2.11 ± 0.02	1.63 ± 0.02	2.07 ± 0.02	1.62 ± 0.03	7.44 ± 0.07
8	1660	2.43 ± 0.02	1.78 ± 0.02	1.99 ± 0.03	1.49 ± 0.03	7.68 ± 0.07

Least square means are shown for the fixed effect levels in the model for analysis of FCS of different body regions (neck back, rump, belly) and total FCS. FCS is feather condition sore, for each body region FCS ranges from 1 through 5, and total FCS ranges from 4 through 20; ^1^only laying house 1.

### Genetic parameters

#### Classical animal model

Table 2 shows the results of the likelihood ratio test. For all lines and body regions, including a random cage effect or a random animal effect improved the goodness of fit significantly (all P-values < 0.001 or < 0.01, respectively).

**Table 2:** Model comparison for analysis of FCS using a likelihood ratio test

		**Cage**^**3**^		**Classical**^**4**^		**Direct-indirect**^**5**^	
**Line**	**Region**	**LR**^**1**^	**P (vs null**^**2**^**)**	**LR**	**P (vs cage**^**3**^**)**	**LR**	**P (vs classical**^**4**^**)**
W1	Neck	604.0	< 0.001	64.8	< 0.001	16.3	< 0.001
	Back	209.2	< 0.001	70.4	< 0.001	22.9	< 0.001
	Rump	1025.4	< 0.001	8.5	0.004	11.5	< 0.001
	Belly	326.2	< 0.001	105.2	< 0.001	4.1	0.128
	Total	862.3	< 0.001	83.7	< 0.001	29.5	< 0.001
WB	Neck	1220.9	< 0.001	156.3	< 0.001	48.9	< 0.001
	Back	1124.8	< 0.001	45.2	< 0.001	66.2	< 0.001
	Rump	1698.9	< 0.001	57.7	< 0.001	66.6	< 0.001
	Belly	401.0	< 0.001	55.8	< 0.001	15.4	< 0.001
	Total	1565.2	< 0.001	132.8	< 0.001	90.7	< 0.001

Model comparison was done for FCS in different regions of the body and for the total FCS in two lines (W1, WB); ^1^LR is the likelihood ratio test (LR is two times the difference in log likelihood between the complex model and the less complex model); ^2^null is a model without random effects; ^3^Cage is a model with only a random cage effect; ^4^Classical is a model with an additive genetic effect and a random cage effect (Equation 1); ^5^Direct-indirect is a model with both direct and indirect genetic effects (Equation 2).

Table 3 and Additional file 1: Table S1 show the estimated genetic parameters obtained with the classical animal model. Using univariate analysis, heritabilities (*h*^2^) for FCS of the different regions and for total FCS were low, ranging from 1.5 to 9.8%. In previous studies, *h*^2^ estimates for total FCS ranged from 22 to 54% [34-36]. To our knowledge, no *h*^2^ estimates for separate body regions have been reported in the literature.

**Table 3:** **Estimates of heritability from classical animal model (*h***^2^** ± SE) and direct–indirect effects model (*T***^2^** ± SE) for FCS**

**Line**	**Region**	$h_{\mathrm{univariate}}^{2}$	$h_{\mathrm{bivariate}}^{2}$	$T_{\mathrm{univariate}}^{2}$	$T_{\mathrm{bivariate}}^{2}$
W1	Neck	0.059 ± 0.015	0.056 ± 0.014	0.195 ± 0.077	0.187 ± 0.074
	Back	0.074 ± 0.018	0.077 ± 0.019	0.257 ± 0.083	0.237 ± 0.077
	Rump	0.015 ± 0.007	0.015 ± 0.007	0.098 ± 0.066	0.094 ± 0.063
	Belly	0.079 ± 0.018	0.077 ± 0.017	0.143 ± 0.061	0.134 ± 0.058
	Total	0.060 ± 0.014	0.060 ± 0.014	0.251 ± 0.089	0.249 ± 0.089
WB	Neck	0.098 ± 0.018	0.102 ± 0.020	0.371 ± 0.117	0.406 ± 0.121
	Back	0.052 ± 0.014	0.048 ± 0.013	0.349 ± 0.110	0.397 ± 0.114
	Rump	0.048 ± 0.012	0.046 ± 0.012	0.456 ± 0.131	0.482 ± 0.133
	Belly	0.063 ± 0.017	0.068 ± 0.017	0.253 ± 0.091	0.255 ± 0.090
	Total	0.095 ± 0.018	0.093 ± 0.017	0.536 ± 0.140	0.588 ± 0.145

Estimates are shown for FCS in each body region and for total FCS in two lines (W1, WB); *h*^2^ is the heritability obtained with the classical animal model using an univariate model ($h_{\mathrm{univariate}}^{2}$) or a bivariate model with mortality ($h_{\mathrm{bivariate}}^{2}$); *T*^2^ is the total heritable variance expressed as a proportion of the phenotypic variance obtained with the direct–indirect effects model using an univariate model ($T_{\mathrm{univariate}}^{2}$) or a bivariate model with mortality ($T_{\mathrm{bivariate}}^{2}$); FCS is feather condition score, for each body region FCS ranges from 1 through 5, and total FCS ranges from 4 through 20.

There are several reasons that could explain the difference in heritabilities between the present and previous studies. In the present study, FCS were recorded when the hens were 40 weeks of age, whereas in previous studies, they were recorded when hens were between 51 and 56 weeks of age [34,36]. Kjaer and Sørensen [36] reported that *h*^2^ of FP behaviour (inflicting and receiving FP) increased when hens grew older (69 weeks compared to 38 weeks). Using younger birds could explain the lower *h*^2^.

A second reason for the lower *h*^2^ observed in this study relates to the use of individual records *vs.* records pooled by cage. We used individual records on four random hens of the same line kept in one cage, while Craig and Muir [34] used the average FCS of cages of three full sibs, which has two effects. First, it averages residuals over cage members, which reduces residual variance ($\sigma_{\bar{e}}^{2}<\sigma_{e}^{2}$) and thus increases heritability. Second, as demonstrated by Peeters et al. [37], the analysis of cage averages yields an estimate of the total heritable variation ($\sigma_{\mathrm{TBV}}^{2}$), rather than of the ordinary (direct) additive genetic variance ($\sigma_{A}^{2}$). Thus, the estimate of Craig and Muir [34] refers to $\sigma_{\mathrm{TBV}}^{2}$ instead of $\sigma_{A}^{2}$. Together, those effects may explain the substantially higher *h*^2^ found by Craig and Muir [34].

#### Direct–indirect effects model

Statistical comparison of the direct–indirect effects model (Equation 2) and the classical animal model (Equation 1), showed a significant improvement of the goodness of fit for both lines and for all body regions (Table 2; all P-values < 0.001 except P = 0.13 for belly in line W1), providing evidence for indirect genetic effects on FCS for almost all body regions.

Table 3 and Additional file 1: Table S2 show the estimated genetic parameters obtained with the direct–indirect effects model. Except for the direct–indirect genetic correlations, most of the genetic parameters were significantly different from zero for both lines. As expected, the standard deviation of the direct breeding value ($\sigma_{A_{D}}$) for the different body regions was of similar magnitude as *σ*_*A*_ from the classical animal model [see Additional file 1: Tables S1 and S2]. The magnitude of $\sigma_{A_{D}}$ is not affected, because groups are composed of non-relatives [37]. Overall, estimates for $\sigma_{A_{D}}$ and $\sigma_{A_{S}}$ were highest in line WB. For all body regions in both lines, the standard deviation of the total breeding value (*σ*_*TBV*_) was 1.3 to 4.0-fold larger than $\sigma_{A_{D}}$, which indicates substantial indirect genetic effects. Again, line WB yielded the highest *σ*_*TBV*_. For the body regions with significant IGE, non-direct genetic effects explained 70 (neck line W1) to 94% (rump line WB) of the total heritable variation in FCS (Table 4). In both lines, the contribution of non-direct genetic effects was highest for the rump region, explaining approximately 93% of the total heritable variation. Using univariate analysis, the total heritable variance expressed as the proportion of phenotypic variance (*T*^2^) ranged from 9.8 to 53.6% (Table 3). Line WB yielded the highest *T*^2^.

**Table 4:** Contribution of direct and indirect genetic effects to total heritable variation of FCS

		**Contribution to the total heritable variation**^**1**^**(%)**			
**Line**	**Region**	**Direct**^**2**^	**Indirect**^**3**^	**Covariance**^**4**^	**Total non-direct**^**5**^
W1	Neck	30.5	56.0	13.5	69.5
	Back	29.4	57.2	13.4	70.6
	Rump	8.4	98.4	-6.8	91.6
	Belly	55.6	34.9	9.4	44.4
	Total	22.0	68.9	9.1	78.0
WB	Neck	20.6	94.8	-15.4	79.4
	Back	7.5	114.7	-22.2	92.5
	Rump	6.3	96.0	-2.3	93.7
	Belly	27.5	50.7	21.8	72.5
	Total	10.1	106.6	-16.7	89.9

Contribution of direct genetic effects (direct), indirect genetic effects (indirect), the covariance between direct and indirect genetic effects (covariance), and total non-direct genetic effects (total non-direct) to total heritable variation of FCS in different body regions for two lines (W1, WB); ^1^Total heritable variation ($\sigma_{\mathrm{TBV}}^{2}=\sigma_{A_{D}}^{2}+2\left(n-1\right)\sigma_{A_{\mathrm{DS}}}+{\left(n-1\right)}^{2}\sigma_{A_{S}}^{2}$; ^2^direct = $\sigma_{A_{D}}^{2}$; ^3^indirect = ${\left(n-1\right)}^{2}\sigma_{A_{S}}^{2}$; ^4^covariance = $2\left(n-1\right)\sigma_{A_{\mathrm{DS}}}$; ^5^total non-direct = $2\left(n-1\right)\sigma_{A_{\mathrm{DS}}}+{\left(n-1\right)}^{2}\sigma_{A_{S}}^{2}$.

In this study, line WB had the lowest FCS, whereas line W1 had the highest FCS. Therefore, it was expected that the contribution of indirect effects would be highest in line WB, because FCS depends on the behaviour of group mates. The estimates of genetic parameters for direct and indirect effects found here were indeed in line with those expectations. Furthermore, estimated genetic parameters were in agreement with the results of Ellen et al. [25], who showed that the total heritable variation in survival time was substantially larger than suggested by the classical animal model. The estimated breeding values obtained in our analysis also provide an elegant way of discriminating between individuals that inflict FP and have high EBV for indirect effects, and individuals that are victims of FP and have high EBV for direct effects, as was previously suggested by Biscarini et al. [16].

#### Bivariate analysis with mortality

Estimating genetic parameters using the bivariate classical animal model and the bivariate direct–indirect effects model, both with mortality (0/1) at 40 weeks of age, did not result in significant changes of the estimated genetic parameters compared to the univariate analyses (Table 3). Comparing log likelihoods of the bivariate model with a model in which the variance components were fixed to the estimates obtained from the univariate analysis, did not change the log likelihoods substantially (data not shown). Therefore, for this dataset, it is not necessary to use bivariate analyses with mortality to estimate genetic parameters for FCS of the different body regions.

#### Genetic correlations between body regions

Estimates of genetic correlations from the direct–indirect effects model are in Table 5 and Additional file 1: Table S3. In both lines, genetic correlations ($r_{12_{T}}$) were positive. The highest estimated genetic correlations were found between adjacent regions, whereas the lowest estimates were found between any region and belly (except for neck-belly in line WB). So far, there are no studies that report genetic correlations for FCS between different body regions. In previous studies, FCS of different body regions were combined and analyses were done on the total FCS [34-36]. In the present study, the high genetic correlations between adjacent regions (neck, back, and rump) suggest that these regions could be combined into a single total FCS. The on average low genetic correlations with belly damage suggest that belly is a distinct trait (except for neck-belly in line WB, which suggests that neck and belly could be combined in a single FCS for this line). FP in the belly region may be closely related with cannibalistic vent pecking. Vent pecking and FP are caused by different internal and external factors [1]. Furthermore, FP is thought to be a redirected foraging behaviour [10], whereas vent pecking seems to be a separate form of damaging behaviour [1,38]. In addition, when comparing the contribution of direct effects to the total heritable variation (Table 4), belly has a larger contribution of direct effects than the other body regions, while the contribution of indirect effects is lower for belly. This could indicate that a different behaviour is associated with pecking on the belly. Therefore, the belly region should be analysed separately and should not be included in the total FCS, as also reported by Parmentier et al. [39]. Furthermore, Bilčik and Keeling [28] showed that feathers of the belly were pulled out more easily, which might overestimate the FCS due to FP.

**Table 5:** Estimates of genetic correlations between total breeding values for FCS

	**Region**			
**Region**	**Neck**	**Back**	**Rump**	**Belly**
Neck		0.81 ± 0.13	NC^1^	0.52 ± 0.24
Back	NC^1^		>0.99	0.34 ± 0.25
Rump	0.87 ± 0.07	0.95 ± 0.04		0.70 ± 0.24
Belly	0.85 ± 0.13	0.72 ± 0.15	0.46 ± 0.19	

Estimates of genetic correlations between total breeding values ($r_{12_{T}}$) for FCS are shown for the four body regions, and for the two lines, line W1 (above diagonal) and line WB (below diagonal); ^1^NC = not converged.

### Effect of person recording FCS

FCS was recorded by four persons, which could introduce bias due to incomplete correlation of FCS scores. The phenotypic correlation between observers was greater than 0.84 for neck, back, and rump, but was as low as 0.64 for belly (data not shown). The largest difference in means of the persons recording FCS was found for neck (for further details see Additional file 1: Table S4). Adams et al. [40] reported a mean correlation of 0.88 between total FCS recorded by three persons.

To investigate the effect of the person recording the FCS on genetic parameters: (1) heterogeneity of residual variance was evaluated by fitting a separate residual variance for person and (2) genetic correlations between persons were estimated using the classical animal model (data not shown). Accounting for heterogeneous residual variances did not change the estimated genetic parameters. Therefore, homogeneous residual variance was assumed. Genetic correlations of total FCS between persons were greater than 0.80 for both lines, which indicates only minor differences in FCS between persons at the genetic level.

### Future breeding program

In this study, we showed that a substantial part of the total genetic variation in plumage condition of different body regions in two purebred layer lines is due to IGE. Accounting for IGE in the genetic analysis showed that total heritable variation was up to 9-fold greater than suggested by results of the classical animal model. Thus, using breeding programs that exploit the heritable variation due to IGE can considerably accelerate response to selection on FCS. In previous studies, FP was significantly related with lower FCS [14,28]. Since FP can result in death (referred to as cannibalism) [2], it is worthwhile to investigate whether FCS at 40 weeks of age can be used as a predictor for survival at the end of the laying period. In this study, we showed that genetic correlations are high between FCS for adjacent body regions, whereas FCS for belly can be considered as a distinct trait, which suggests that neck, back and rump can be combined into a total FCS. However, before drawing this conclusion, it is important to investigate whether total FCS, total FCS (without belly), or separate body regions can be used as predictor for survival at the end of the laying period. This could contribute to reducing an important welfare problem in laying hens. Regardless, both breeding and management solutions should be applied to prevent FP.

Measuring the plumage condition by recording FCS is time-consuming. Moreover, laying hens need to be at least 40 weeks before FCS can be recorded, and FCS measured on selection candidates is not very useful, because selection candidates are housed individually. Hence, selection would have to be based on sib or progeny information. These obstacles can be overcome by using genomic selection [41,42]. Our results clearly show that genetic improvement of plumage condition cannot rely on DGE only. Therefore, genomic selection methods must be extended to accommodate IGE. A challenge is how to design a reference population that is suitable for genomic selection on plumage condition and survival time in laying hens.

## Conclusions

Social interactions have a substantial effect on plumage condition in laying hens. This study shows that, for the different body regions (neck, back, rump, and belly), the total heritable variance of FCS, expressed as the proportion of the phenotypic variance (*T*^2^) ranges from 9.8 (rump line W1) to 53.6% (total FCS). A substantial part, 70 to 94%, of the total heritable variation relates to IGE. Thus, it is expected that including both direct and indirect effects in a genetic selection program will contribute to a reduction in FP behaviour, one of the major welfare issues in the laying hen industry.

## Competing interests

The authors declare that they have no competing interests.

## Authors’ contributions

TB performed data analysis, wrote the first draft of the manuscript. PB was the principal supervisor of the study and assisted with data analysis and preparation of the manuscript. JV was in charge of the experiment and reviewed the manuscript. TBR helped with the experiment and reviewed the manuscript. EDE performed data analysis, wrote and prepared the manuscript for submission. All authors read and approved the manuscript.

## Supplementary Material

Additional file 1: Table S1 Title: Estimates of genetic parameters from the univariate classical animal model and corresponding SE of FCS. Description: Data represents estimates of genetic parameters from the univariate classical animal model and corresponding SE of FCS for each body region and the total FCS in the two lines (W1, WB). **Table S2.** Title: Estimates of genetic parameters from the univariate direct–indirect effects model and corresponding SE of FCS. Description: Estimates of genetic parameters for direct and indirect effects for FCS in each body region and for total FCS in the two lines (W1, WB). **Table S3.** Title: Estimates of genetic correlations between the four body regions from the direct–indirect effects model. Description: Estimates are shown for the direct genetic correlations ($r_{12_{D}}$; below the diagonal) and indirect genetic correlations ($r_{12_{S}}$; above the diagonal) between the four body regions in two lines (W1, WB) from the direct–indirect effects model. **Table S4.** Title: Means with corresponding SD of the four persons who recorded the FCS. Description: Means of the four persons who recorded the FCS for each body region in two lines (W1, WB). Click here for file
