# Supplementary material for: Plumage condition in laying hens: genetic parameters for direct and indirect effects in two purebred layer lines
Source: Genet Sel Evol. 2014 May 29;46(1):33. doi: 10.1186/1297-9686-46-33 (PMC4073196; doi:10.1186/1297-9686-46-33)
Supplement: Additional file 1: Table S1 — Title: Estimates of genetic parameters from the univariate classical animal model and corresponding SE of FCS. Description: Data represents estimates of genetic parameters from the univariate classical animal model and corresponding SE of FCS for each body region and the total FCS in the two lines (W1, WB). Table S2. Title: Estimates of genetic parameters from the univariate direct–indirect effects model and corresponding SE of FCS. Description: Estimates of genetic parameters for direct and indirect effects for FCS in each body region and for total FCS in the two lines (W1, WB). Table S3. Title: Estimates of genetic correlations between the four body regions from the direct–indirect effects model. Description: Estimates are shown for the direct genetic correlations (r12D; below the diagonal) and indirect genetic correlations (r12S; above the diagonal) between the four body regions in two lines (W1, WB) from the direct–indirect effects model. Table S4. Title: Means with corresponding SD of the four persons who recorded the FCS. Description: Means of the four persons who recorded the FCS for each body region in two lines (W1, WB). [file 1297-9686-46-33-S1.pdf]

Additional file 1

Table S1 Estimates of genetic parameters from the univariate classical animal model and corresponding SE of FCS

Line	Region	σ_A	σ^2_{cage}	σ^2_P
W1	Neck	0.134±0.017	0.099±0.006	0.307±0.007
	Back	0.117±0.015	0.035 ±0.003	0.186±0.004
	Rump	0.059±0.014	0.105 ±0.005	0.243±0.006
	Belly	0.180±0.021	0.099±0.007	0.414±0.009
	Total	0.348±0.043	0.805 ±0.044	2.031±0.048
WB	Neck	0.307±0.029	0.471 ±0.023	0.956±0.025
	Back	0.221±0.030	0.435±0.022	0.933±0.023
	Rump	0.238±0.030	0.680±0.031	1.184±0.032
	Belly	0.229±0.031	0.235±0.016	0.831±0.019
	Total	0.949±0.091	5.260±0.241	9.479±0.257

Estimates are shown for FCS in each body region and for total FCS in two lines (W1, WB); σ_A is the genetic standard deviation; σ_c^2 is the cage variance; σ_P^2 is the phenotypic variance: $\sigma_P^2 = \sigma_A^2 + \sigma_c^2 + \sigma_e^2$. FCS is feather condition score, for each body region FCS ranges from 1 through 5, and total FCS ranges from 4 through 20.

Table S2 Estimates of genetic parameters from the univariate direct-indirect effects model and corresponding SE of FCS

Line	Region	σ_{A_D}	σ_{A_S}	σ_{TBV}	σ^2_{cage}	σ^2_P	r_A
W1	Neck	0.135 ±0.019	0.061 ±0.013	0.244 ±0.049	0.087 ±0.007	0.306 ±0.007	0.16 ±0.23
	Back	0.118 ±0.015	0.055 ±0.010	0.218 ±0.036	0.026 ±0.004	0.185 ±0.004	0.16 ±0.21
	Rump	0.045 ±0.019	0.051 ±0.013	0.154 ±0.052	0.100 ±0.006	0.243 ±0.006	-0.12 ±0.51
	Belly	0.181 ±0.021	0.048 ±0.016	0.243 ±0.052	0.092 ±0.009	0.414 ±0.009	0.11 ±0.28
	Total	0.335 ±0.047	0.197 ±0.033	0.713 ±0.129	0.701 ±0.053	2.022 ±0.049	0.12 ±0.21
WB	Neck	0.270 ±0.031	0.193 ±0.026	0.595 ±0.097	0.403 ±0.029	0.954 ±0.026	-0.17 ±0.18
	Back	0.157 ±0.032	0.204 ±0.025	0.572 ±0.092	0.367 ±0.027	0.936 ±0.025	-0.38 ±0.24
	Rump	0.185 ±0.035	0.240 ±0.030	0.735 ±0.110	0.559 ±0.040	1.185 ±0.034	-0.05 ±0.22
	Belly	0.241 ±0.033	0.109 ±0.022	0.459 ±0.084	0.197 ±0.021	0.832 ±0.019	0.29 ±0.22
	Total	0.718 ±0.096	0.775 ±0.084	2.254 ±0.309	4.170 ±0.318	9.475 ±0.276	-0.25 ±0.18

Estimates are shown for FCS in each body region and for total FCS in two lines (W1, WB); σ_{A_D} and σ_{A_S} are estimates of the standard deviation of the direct breeding value and the standard deviation of the indirect breeding value. σ_{TBV} is the standard deviation of the total breeding value: $\sigma^2_{TBV} = \sigma^2_{A_D} + 2(n-1)\sigma_{A_{DS}} + (n-1)^2\sigma^2_{A_S}$. σ^2_c is the cage variance. σ^2_P is the phenotypic variance: $\sigma^2_P = \sigma^2_{A_D} + (n-1)\sigma^2_{A_S} + \sigma^2_c + \sigma^2_e$. r_A is the genetic correlation between the direct breeding value and the indirect breeding value. FCS is feather condition score, for each body region FCS ranges from 1 through 5, and total FCS ranges from 4 through 20.

Table S3 Estimates of genetic correlations between the four body regions from the direct-indirect effects model.

		Region			
**Line**	**Region**	**Neck**	**Back**	**Rump**	**Belly**
W1	Neck		0.84±0.13	NC[1]	>0.99
	Back	0.75±0.11		0.92±0.14	0.75±0.30
	Rump	NC[1]	0.87±0.20		>0.99
	Belly	0.09±0.18	-0.02±0.18	0.48±0.28	
WB	Neck		NC[1]	0.97±0.03	0.92±0.09
	Back	NC[1]		0.98±0.03	0.85±0.11
	Rump	0.65±0.14	0.74±0.13		0.74±0.13
	Belly	0.47±0.15	0.46±0.20	0.25±0.21	

Estimates are shown for FCS in each body region in two lines (W1, WB); the estimates below the diagonal refer to the direct genetic correlations ($\boldsymbol{r_{12_D}}$); the estimates above the diagonal refer to the indirect genetic correlations ($\boldsymbol{r_{12_S}}$); [1]NC = not converged

Table S4 Means with corresponding SD of the four persons who recorded the FCS

Person	n	W1 Neck	Back	Rump	Belly	WB Neck	Back	Rump	Belly
1	2988	1.30 ±0.59	1.30 ±0.41	1.19 ±0.52	1.36 ±0.70	2.28 ±1.18	1.60 ±1.04	2.17 ±1.25	1.72 ±1.01
2	2870	1.22 ±0.47	1.12 ±0.40	1.23 ±0.57	1.32 ±0.75	1.99 ±1.01	1.70 ±1.10	2.26 ±1.19	1.57 ±0.99
3	2970	1.48 ±0.68	1.16 ±0.46	1.13 ±0.46	1.36 ±0.67	2.65 ±1.21	1.68 ±1.11	2.13 ±1.30	1.65 ±0.96
4	3364	1.46 ±0.57	1.22 ±0.48	1.16 ±0.48	1.17 ±0.50	2.32 ±1.11	1.63 ±0.96	2.11 ±1.26	1.49 ±0.82

Estimates with corresponding SD are shown for each body region in two lines (W1, WB); FCS is feather condition score, for each body region FCS ranges from 1 through 5, and total FCS ranges from 4 through 20.
